# Non-lytic expulsion/exocytosis of *Candida albicans* from macrophages

**DOI:** 10.1016/j.fgb.2012.01.008

**Published:** 2012-09

**Authors:** Judith M. Bain, Leanne E. Lewis, Blessing Okai, Janet Quinn, Neil A.R. Gow, Lars-Peter Erwig

**Affiliations:** a Immunology and Infection, School of Medicine and Dentistry, University of Aberdeen, Aberdeen AB25 2ZD, UK; b Institute for Cell and Molecular Biosciences, Faculty of Medical Sciences, Newcastle University, Newcastle upon Tyne NE2 4HH, UK; c Aberdeen Fungal Group, School of Medical Sciences, University of Aberdeen, Aberdeen AB25 2ZD, UK

**Keywords:** *Candida albicans*, Macrophage, Phagocytosis, Innate immunology, Fungal infection

## Abstract

*Candida albicans* is an opportunistic pathogen and is recognised and phagocytosed by macrophages. Using live-cell imaging, non-lytic expulsion/exocytosis of *C. albicans* from macrophages is demonstrated for the first time. Following complete expulsion, both the phagocyte and pathogen remain intact and viable. Partial engulfment of hyphal *C. albicans* without macrophage lysis is also demonstrated. These observations underpin the complexity of interactions between *C. albicans* and innate immune cells.

## 1 Introduction

*Candida albicans* inhabits the human mucosa as a commensal fungus, however superficial and systemic lethal disease can arise under circumstances of perturbed host susceptibility. Associated with its infectious capacity is a repertoire of virulence attributes, of which the dimorphic transition between budding yeast and hypha is key. This facilitates niche-specific growth, tissue penetration, dissemination and immune evasion/escape (Kumamoto and Vinces, 2005). Host defence against systemic candidiasis relies mainly on the ingestion and elimination of fungal cells by cells of the innate immune system, especially neutrophils and macrophages (Sheth et al., 2011; McKenzie et al., 2010). Our recent work carefully describes the phagocytic process during interactions of macrophages with *C. albicans* using sophisticated video microscopy and tracking (Lewis et al., in press). We and others have shown that engulfment of live hyphal-forming strains leads ultimately to host cell lysis and death (McKenzie et al., 2010; Lo et al., 1997). May, Casadevall and colleagues have previously observed yeast cells of *Cryptococcus neoformans* (Ma et al., 2006; Alvarez and Casadevall, 2006; Johnston and May, 2010) undergoing expulsion from phagocytes, rendering both host and pathogen viable. We report here non-lytic expulsion/exocytosis as an alternative outcome following phagocytosis of hyphae-producing *C. albicans* by the murine macrophage cell line J774.1. We show that both the fungal pathogen and the macrophage remain viable and functional (retain phagocytic ability/continued hyphal growth) after non-lytic expulsion. This is not only the first demonstration of non-lytic expulsion for a *Candida* species but also the first time this was observed in the context of hyphal cells.

## 2 Description of observations

Hyphal-mediated macrophage lysis was captured using live cell imaging (Video 1). The central macrophage internalizes *C. albicans* and we observed subsequent extension of hyphal filaments within the macrophage (Video 1). In a majority of cases, the membranes of the macrophage ultimately failed to restrain hyphal expansion; resulting in the eventual rupture of the phagocyte (Video 1). *C. albicans* escape and associated macrophage lysis are common features of *C. albicans*-macrophage interactions and have been studied in more detail previously (Sheth et al., 2011).

However, in a minority of cases, non-lytic expulsion/exocytosis was observed (Video 2). Although this occurred at a very low frequency (<1%, *n* > 50) it was observed repeatedly in various experimental conditions. The video shows that the central macrophage (stained red) engulfing a yeast cell *C. albicans* (stained green) followed by hyphal growth inside the macrophage. Fungal expulsion proceeded with the hypha emitted tip-first and was completed within approximately 40 min (Video 2). Following externalisation of *C. albicans*, both macrophage and fungus remained viable – the macrophage proceeded to undergo mitosis to yield motile daughter cells and the hypha continued to extend at normal rates (Video 2).

We also report partial engulfment of hyphal *C. albicans* without macrophage lysis (Video 3). In the example, a yeast cell was engulfed leaving the growing hypha exposed; this hypha was then engulfed by a second macrophage (Video 3). Extensive stretching of the host cell ensued, after which the macrophage plasma membrane retracted back from the growing hypha (Video 3). In contrast to macrophage lysis resulting from hyphal penetration of the membrane (Video 1), the host cell remained viable and active (Video 3). While a portion of the fungus remained within the macrophage, the externalised hyphal tip was later recognised and engulfed by a third macrophage as it continued to grow (Video 3).

## 3 Discussion

Here we report non-lytic expulsion/exocytosis of *C. albicans* from macrophages, the first demonstration of this process for a filamentous fungus. This process has been described previously for the facultative pathogenic yeast *C. neoformans* (Ma et al., 2006; Alvarez and Casadevall, 2006). Studies suggest that non-lytic expulsion, or ‘vomocytosis’ of *C. neoformans* occured in 7–10% of J774.1 in a 24 h period, although with greater frequency in primary cells and in vivo (Lo et al., 1997; Ma et al., 2006; Alvarez and Casadevall, 2006). Our in vitro system detected non-lytic complete expulsion of *C. albicans* at a frequency of <1% within a 6 h period, after which the majority of macrophages were overcome by significant hyphal growth in the culture medium. Even though non-lytic complete expulsion of *C. albicans* is a rare event, that is difficult to quantify, it is not an artefact and was observed repeatedly (*n* > 50) with control strains and glycosylation-deficient mutant strains under a range of experimental conditions. To date we have only observed non-lytic expulsion in macrophage cell lines and not in primary mouse or human macrophages but this may simply be a reflection of the limited number of interactions investigated in primary cells to date.

Increase of phagosomal pH increases the rate of *C. neoformans* vomocytosis from macrophages (Nicola et al., 2011). Both hyphal and yeast forms of *C. albicans* are know to modify the phagosomes in which they are internalised by raising pH (Fernandez-Arenas et al., 2009), therefore the occurrence and frequency of non-lytic expulsion of *C. albicans* may also be related to intraphagosomal pH.

The clinical relevance of this phenomenon is difficult to determine and warrants further investigation as non-lytic expulsion of hyphal *C. albicans* may benefit the macrophage in terms of avoiding lysis, but conversely may also benefit the fungus as it escapes the microbicidal environment of the phagosome. This study underpins the complexities of the host/fungus interaction of a species which has both commensal and infectious capacity.

## 4 Methods

*C. albicans* strain (NGY152) hitherto referred to as the “control” strain was grown in SC-Ura at 30 °C and J774.1 macrophages were cultured in supplemented DMEM medium at 37 °C with 5% CO_2_, as described previously (McKenzie et al., 2010). For phagocytosis assays, 1 × 10^6^ macrophages were seeded to glass based dishes and cultured overnight at 37 °C with 5% CO_2_. During experiments, medium was replaced with supplemented CO_2_-independent medium containing 1 μM Lysotracker Red (Invitrogen, Paisley, UK) (Erwig et al., 2006). *C. albicans were* stained with 1 mg/ml FITC cell membrane label (Sigma, UK) following manufactures instruction, in 0.05 M carbonate-bicarbonate buffer (pH 9.6) for 10 min at 20 °C in the dark, washed three times and resuspended in PBS, were added to macrophages at a 3:1 ratio. Video microscopy was conducted at 37 °C with a DeltaVision Core microscope (Applied Precision, Washington, USA) and images captured at 1 min intervals for 6 h by an EMCCD camera.
