# Supplementary figures and images for: Non-lytic expulsion/exocytosis of Candida albicans from macrophages
Source: Fungal Genet Biol. 2012 Sep;49(9):677–8. doi: 10.1016/j.fgb.2012.01.008 (PMC3430864; doi:10.1016/j.fgb.2012.01.008)

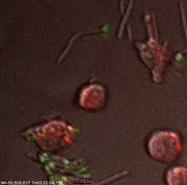

Supplement: Supplementary video 2 [file mmc2.jpg]

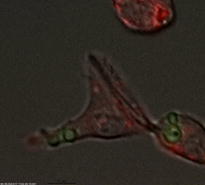

Supplement: Supplementary video 3 [file mmc3.jpg]
